# Transanal Minimally Invasive Surgery (TAMIS) to Treat Vesicorectal Fistula: A New Approach

**DOI:** 10.1590/S1677-5538.IBJU.2014.0002

**Published:** 2015

**Authors:** Marcos Tobias-Machado, Pablo Aloisio Lima Mattos, Leonardo Oliveira Reis, César Augusto Braz Juliano, Antonio Carlos Lima Pompeo

**Affiliations:** 1 Programa de Cirurgia Urológica Minimamente Invasiva, Departamento de Urologia, Faculdade de Medicina do ABC, Santo André, São Paulo, Brasil; 2 Seção de Uro-oncologia, Departamento de Urologia, Faculdade de Medicina do ABC, Santo André, São Paulo, Brasil; 3 Departamento de Urologia, Faculdade de Medicina do ABC, Santo André, São Paulo, Brasil; 4 Divisão de Urologia da Faculdade de Ciências Médicas da Universidade de Campinas, UNICAMP, Campinas, Brasil; 5 Faculdade de Medicina - Divisão de Urologia do Centro de Ciências da Vida, Pontifícia Universidade Católica de Campinas (PUC-Campinas), Brasil

**Keywords:** Fistula, Postoperative Complications, Natural Orifice Endoscopic Surgery

## Abstract

**Purpose:**

Vesicorectal fistula is one of the most devastating postoperative complications after radical prostatectomy. Definitive treatment is difficult due to morbidity and recurrence. Despite many options, there is not an unanimous accepted approach. This article aimed to report a new minimally invasive approach as an option to reconstructive surgery.

**Materials and Methods:**

We report on Transanal Minimally Invasive Surgery (TAMIS) with miniLap devices for instrumentation in a 65 year old patient presenting with vesicorectal fistula after radical prostatectomy. We used Alexis® device for transanal access and 3, 5 and 11 mm triangulated ports for the procedure. The surgical steps were as follows: cystoscopy and implant of guide wire through fistula; patient at jack-knife position; transanal access; Identification of the fistula; dissection; vesical wall closure; injection of fibrin glue in defect; rectal wall closure.

**Results:**

The operative time was 240 minutes, with 120 minutes for reconstruction. No perioperative complications or conversion were observed. Hospital stay was two days and catheters were removed at four weeks. No recurrence was observed.

**Conclusions:**

This approach has low morbidity and is feasible. The main difficulties consisted in maintaining luminal dilation, instrumental manipulation and suturing.

## INTRODUCTION

Vesicorectal fistula consists of an abnormal communication between the bladder urothelium and rectal mucosa, which represents a devastating condition. Diverticulitis, Crohn's disease and cancer are the most common etiologies (1–5). Vesicorectal fistula is an extremely rare complication following radical prostatectomy.

Patients may present with irritative urinary symptoms, urinary tract infection, pneumaturia, fecaluria and tenesmus (1, 6, 7). Cystoscopy and tomography are the most accurate tests to confirm the diagnosis (1, 7). Non- surgical watchful waiting is an option in selected cases (8). Drug therapy such as antibiotics, intravenous total parenteral nutrition and bowel rest may be used in patients with few symptoms and non-toxemic and non-malignant etiology (9). Proximal colostomy and urinary catheterization are options in poorly responsive or very symptomatic patients. Definitive treatment aiming to separate the organs and close the defect with preservation of function is recommended in the absence of infection or obstruction. Partial resection and interposition of omentum between suture lines may be required. It can be done in stages. (1, 7, 10).

Definitive treatment is challenging due to the morbidity of the classic techniques and the high recurrence rate. Currently we prefer traditional approaches like transanal, transabdominal, trans-sphincteric and transperineal (11). However, there is not an universally accepted approach. Techniques described for minimally invasive repair such as laparoscopic transperitoneal approach can reduce the morbidity of treatment and was recently described with good results (12–14).

These evidences have motivated us to evaluate new approaches as options to treat vesicorectal fistulas in selected cases. This article aims to describe and evaluate the results of a new minimally invasive approach to treat vesicorectal fistula.

## MATERIALS AND METHODS

A 65 year old patient developed a vesico-rectal fistula in the first 20 days after radical prostatectomy to treat prostate cancer. The diagnosis was confirmed by cystoscopy and CT scan, which showed the fistula in the trigone. Conservative treatment was attempted with high absorption diet, suprapubic cystostomy and proximal colostomy, but the treatment failed after two months.

We performed Transanal Minimally Invasive Surgery (TAMIS) with miniLap devices for instrumentation. Initially, the patient was positioned in lithotomy and cystoscopy was made for implant of a hydrophilic guide wire through fistula to facilitate identification and dissection. We did not position ureteral catheters but it can be made to improve safety.

**Figure 1:**
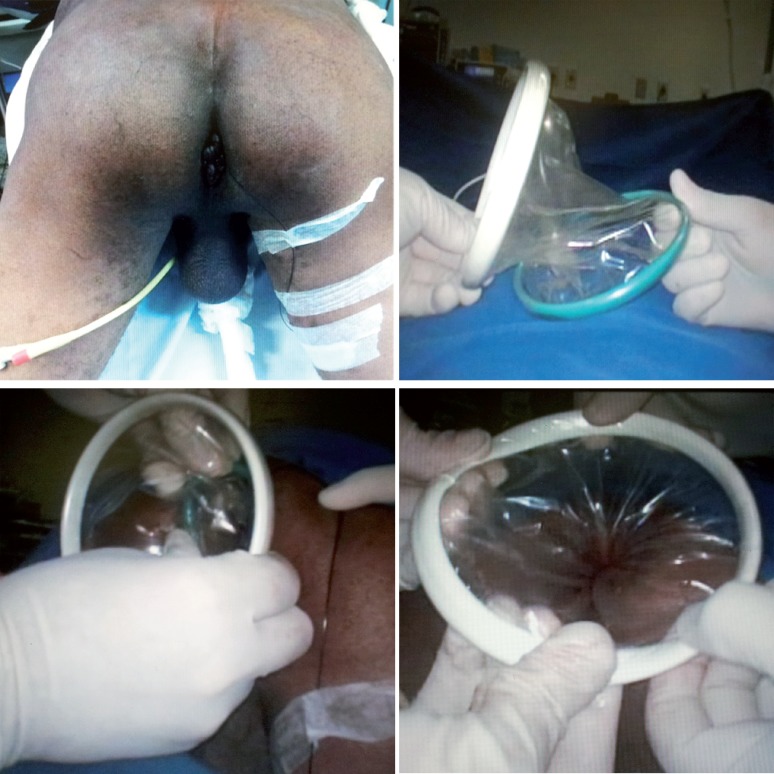
Transanal access by Alexis® device.

The patient was placed in the jack-knife position with the buttocks apart. We used Alexis® (Applied Medical System) device for 2.5-6 cm size incisions to configure the TAMIS platform. The device was inserted into the anal canal and rectum. A silicone glove was fixed in the outer ring of the device and the self-retaining design held the anal canal open, allowing access to the operative field (Figure-1). We positioned three triangulated ports (one 11 mm port for rigid endoscope 0 degrees, one 5 mm port for ultrasonic scalpel and one 3 mm port for minilap devices). We kept the pneumorectum around 15 mmHg.

**Figure 2:**
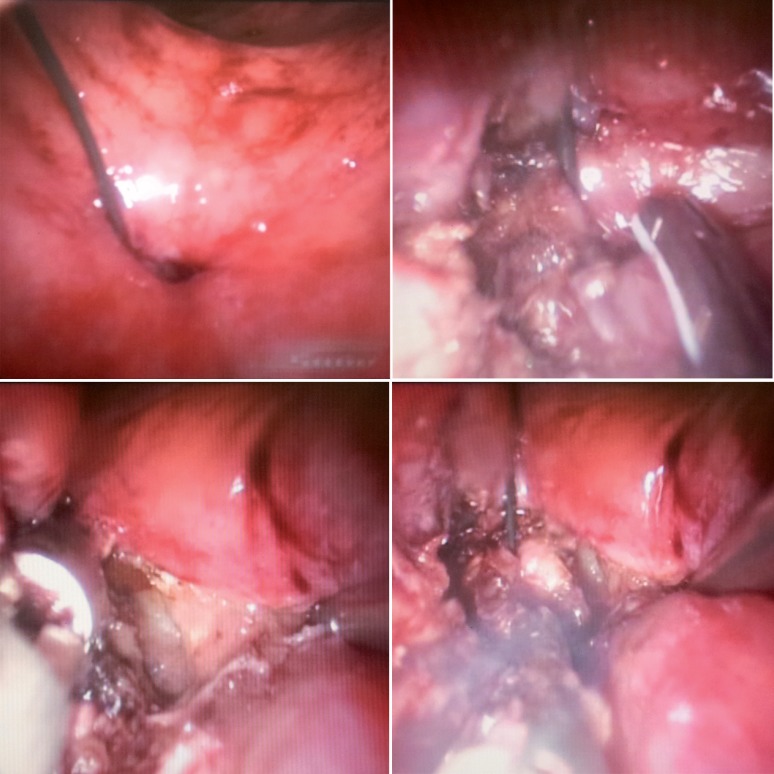
Vesicorectal fistula is excised.

**Figure 3:**
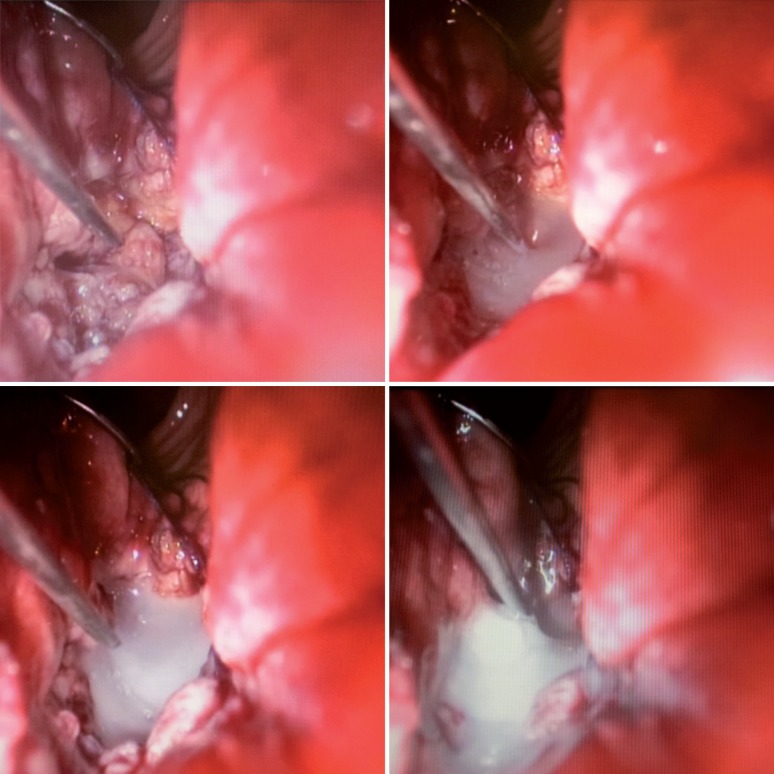
Injection of fibrin glue between vesical and rectal wall.

**Figure 4:**
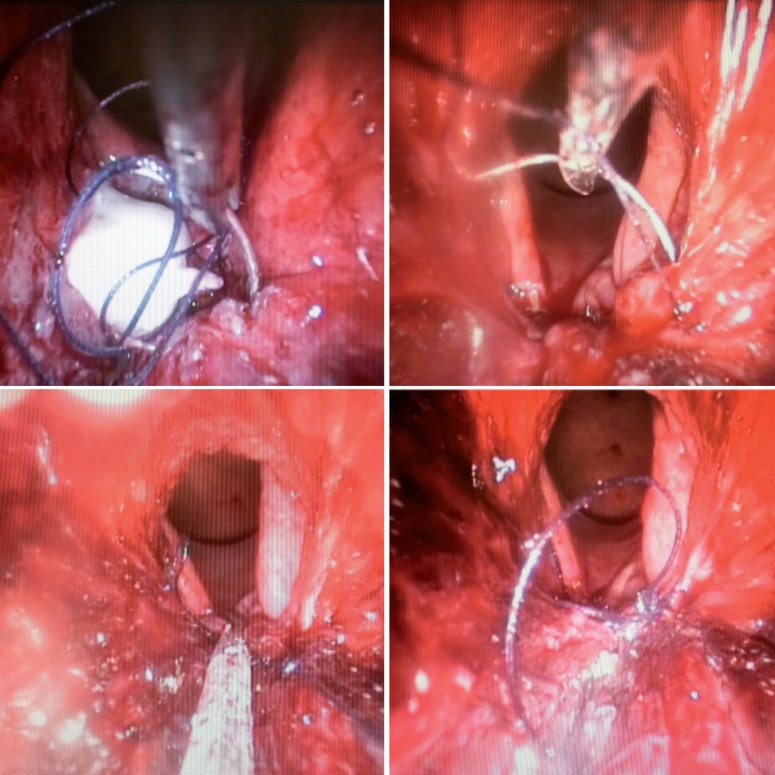
Rectal wall closed.

The fistula was identified 5 cm from the anal verge. The fistula tract was excised with ultrasonic scalpel. The guidewire was removed only at the end of excision (Figure-2). The bladder wall was closed with 3-0 Vicryl in a running suture. The space between the bladder wall and rectal wall was filled with fibrin glue (Figure-3). The rectal wall was closed with 3-0 Vicryl in a running suture (Figure-4). Finally we maintained a urethral catheter 18 Fr and cystostomy 20 Fr.

## RESULTS

The operative time was 240 minutes for TAMIS with 120 minutes for reconstruction.

There was no conversion, perioperative or postoperative complications in the procedure, including bleeding and rectal perforation. The hospital stay was two days.

The full return to daily activities was in two weeks. Cystoscopy was performed after four weeks of surgery and it revealed no signs of fistula. After four months of follow-up no recurrence was observed. There was no stenosis of anal canal and patient is defecating and voiding normally.

## DISCUSSION

There is no consensus about the better approach to treat recto-urinary fistula. Conventional open surgery remains the preferred choice, however it has some limitations (15–17). Trans-abdominal and posterior approaches have the disadvantages of large incisions to achieve good access. Transperineal and transanal approaches are less invasive but limited visualization and small working space can difficult a good repair. Laparoscopic transperitoneal surgery was introduced in 2003. With an excellent magnification the posterior bladder wall is opened including the fistula tract to achieve good visualization and dissection. The advantages are fast recovery with preliminary good results (14). The refinement of this technique using robotic assistive technology was also described with good results (18).

In the current decade, transluminal procedures have been developed to manage selected cases of urologic pathologies. The potential advantages include less effects of carbonic gas, no mobilization or contact with other organs and reduction of entrance ports. The main disadvantages consist in the small working space that requires more skills to reconstructive procedures. Transvesicoscopic surgery has been reported to treat bladder foreign body, lithiasis, diverticula, ureteral reimplantation and vesicovaginal fistula with excellent results (19–21).

Transanal Minimally Invasive Surgery (TAMIS) is a variant of Natural Orifice Endoscopic Surgery (NOES). Presented in 2009, this surgical platform uses access devices that traditionally are used for single site laparoscopy. The most used devices are the SILS™ Port, Alexis Wound Protector/ Retractor and the GelPOINT Path Trans anal Access Platform. The chosen device is inserted into the rectum. When pneumorectum is established, the surgical field is then increased considerably and gives to the surgeon the ability to expand their skills to include procedures from the distal rectum to the mid and proximal rectum. This platform uses standard laparoscopic or minilap instrumentation.

TAMIS was initially described for treatment of benign lesions. After that, treatment of malignant lesions was also described. We observed a growing acceptance in the use of TAMIS to approach anorectal fistulas and tumors at early grades with good results (22–24). Albert and cols. performed a retrospective analysis of 50 patients with benign and malignant rectal lesions treated with TAMIS at a tertiary referral center. All procedures were made without conversion to other approaches and 68% of patients were discharged on the day of surgery. Only 6% were found to have microscopically positive margins. No long-term complications were observed.

TAMIS platform is versatile and there are some applications beyond local excision. There are descriptions of rectourethral fistula repair, ligation of rectal Dieulafoy's lesion and extraction of foreign body. Atallah and cols. performed TA-MIS to treat a man with rectourethral fistula after cryoablation treatment for prostate cancer. A follow-up enema demonstrated resolution of the fistula (23). Gómez et al. comment in a letter to the editor of Actas Urologicas Espanõlas (in press) one case in which TAMIS was utilized to repair an uretrorectal fistula using Gelpoint device. The procedure was successful, achieving a good exposition to 2-layer repair and hemostasis, according to the authors (25).

These works motivated us to propose this new approach to treat vesicorectal fistula and evaluate its results. In our report the duration of surgery was 240 minutes, with 120 minutes for reconstruction. We believe that the limited experience with access and non-availability of a material specifically developed for these new approaches is still an obstacle to overcome and make surgery times prolonged when compared to conventional invasive procedures.

In our procedure the greatest difficulties were maintaining luminal dilation, the instrumental manipulation and intraoperative suture. Nevertheless, the length appears to be similar to trans-peritoneal laparoscopic approaches already described. No complications were observed. The procedure was completed without conversion, and intraoperative bleeding was negligible.

Despite the limitations, careful magnified dissections and subsequent repairs were the elements that allowed a better control and a minimized risk of perioperative complications and conversion.

One of the most feared troubles in the repair of vesicorectal fistulas is the loss of functionality due to rectal morbidity of most techniques traditionally used, with the emergence of problems such as anal stenosis and fecal incontinence. None of these postoperative complications were observed in our report, even with little experience with the new method. Minimally invasive surgery done by an expert professional is less aggressive, reduces the risk of complications and may reproduce the results of traditional techniques.

The length of hospital stay in our report was 2 days. This result was even slightly better than some series in the literature and reinforces the potential of minimally invasive surgery in decreasing the morbidity (14,18,26,27). Although with a short follow-up, the preliminary result is encouraging and shows compliance with the findings in the literature (14,26).

## CONCLUSIONS

Transanal Minimally Invasive Surgery (TAMIS) to treat vesicorectal fistula is feasible and seems to have lower morbidity when compared with more traditional techniques. It is effective and can be offered as an option by experienced laparoscopic surgeons to patients. The greatest difficulties were maintaining luminal dilation, instrumental manipulation and intraoperative suture.

## ABBREVIATIONS

TAMIS: = Transanal Minimally Invasive Surgery.
NOES: = Natural Orifice Endoscopic Surgery.
